# Culture-Based Cervicovaginal Findings and *Candida* Colonization in Third-Trimester Pregnancy: A Prospective Observational Study

**DOI:** 10.3390/medicina62071389

**Published:** 2026-07-18

**Authors:** Ayşe Hazırbulan, Yusuf Üstün, Mihriban Yücel

**Affiliations:** 1 Department of Obstetrics and Gynecology, Ankara Training and Research Hospital, University of Health Sciences, 06230 Ankara, Türkiye; 2 Department of Clinical Microbiology, Ankara Training and Research Hospital, University of Health Sciences, 06230 Ankara, Türkiye

**Keywords:** pregnancy trimester, third, *Candida*, microbiological techniques, vagina, cervix uteri, *Streptococcus agalactiae*

## Abstract

*Background and Objectives*: Culture-based cervicovaginal assessment in late pregnancy may reveal bacterial and fungal findings beyond routine Group B *Streptococcus* (GBS) detection; however, third-trimester non-GBS findings remain insufficiently characterized. This study aimed to describe the distribution of culture-based cervicovaginal findings and to explore the associations between culture-confirmed *Candida* colonization and maternal, obstetric, and microscopic parameters. *Materials and Methods*: This prospective observational study included 300 third-trimester pregnant women at ≥28 gestational weeks who were receiving routine antenatal care at a tertiary center. Cervicovaginal samples were obtained using sterile swabs and analyzed with conventional culture-based methods. Findings were categorized according to the identified microorganisms, and GBS positivity was recorded. For *Candida*-focused analyses, only samples reported as fecal contamination were excluded; women with GBS-positive cervicovaginal cultures were retained in the evaluable cohort. Associations were evaluated using the Mann–Whitney U test and Fisher’s exact test. *Results***:** The mean maternal age was 26.48 ± 5.67 years. Normal vaginal flora was observed in 252 women (84.0%). *Candida* colonization was the most frequent non-GBS finding, identified in 35 women (11.7%), whereas GBS was detected in seven women (2.3%). Other non-GBS bacterial isolates and fecal contamination were reported in four women (1.3%) and two samples (0.7%), respectively. Clue cells, direct microscopic yeast positivity, and *Trichomonas vaginalis* were identified in 20 women (6.7%), 12 women (4.0%), and 6 women (2.0%), respectively. After excluding samples reported as fecal contamination, 298 evaluable cervicovaginal cultures were included in the Candida-focused analysis. Direct microscopic yeast detection was more frequent among women with culture-confirmed Candida colonization than among those without colonization (14.3% vs. 2.3%, *p* = 0.005), whereas Candida colonization was not associated with maternal age, gravidity, parity, gestational age, clue cell positivity, *Trichomonas vaginalis* positivity, or GBS-positive cervicovaginal culture. *Conclusions***:** In third-trimester pregnancy, culture-based cervicovaginal assessment showed that *Candida* colonization was the most prominent non-GBS finding. The higher detection rate of culture-confirmed Candida colonization compared with direct microscopic yeast positivity suggests that microscopy alone may underestimate fungal colonization. These findings suggest that reporting non-GBS culture-based cervicovaginal findings may provide additional descriptive microbiological information beyond routine GBS detection in late pregnancy; however, they should not be interpreted as evidence of clinical outcome associations.

## 1. Introduction

The cervicovaginal environment during pregnancy represents a dynamic microbial ecosystem comprising diverse bacterial and fungal microorganisms [1,2,3]. Pregnancy-related hormonal and immunological changes may influence the composition of the vaginal microbial flora [3]. The vaginal microbial flora is generally dominated by *Lactobacillus* species but may also include other microorganisms in varying proportions [1,4]. This balanced ecosystem contributes to reproductive tract health, whereas disruption of the normal vaginal flora has been associated with alterations in the local microbial environment and may contribute to maternal vaginal symptoms or discomfort [5,6]. Late-pregnancy microbiological assessment has traditionally focused on Group B *Streptococcus* (GBS), as it is an important cause of early-onset neonatal infection. Maternal genitourinary and gastrointestinal colonization represent the major risk factors for vertical transmission, and guideline-recommended GBS screening has been established because of the association between GBS colonization and early-onset neonatal sepsis, as well as the effectiveness of intrapartum antibiotic prophylaxis [7,8]. However, culture-based cervicovaginal assessment may also reveal non-GBS findings, which remain less well-characterized, particularly during the third trimester [9]. Although the role of GBS in pregnancy has been extensively investigated, non-GBS cervicovaginal microorganisms have received relatively little attention. Most available studies have focused on early pregnancy, symptomatic women, or specific clinical conditions, thereby limiting the characterization of culture-based non-GBS cervicovaginal findings in routine late-pregnancy cohorts [10]. Among non-GBS organisms, *Candida* species are frequently detected during pregnancy, although the reported prevalence varies according to population characteristics, gestational age, symptom status, and diagnostic method [11]. Vulvovaginal candidiasis is a common clinical condition; however, *Candida* colonization may also be identified in asymptomatic pregnant women, and its contribution to culture-based cervicovaginal findings in late pregnancy remains insufficiently defined [11]. Other non-GBS findings, including *Gardnerella vaginalis*, aerobic bacterial isolates, clue cells, and microscopic yeast elements, may also be encountered during routine cervicovaginal assessment [12]. Thus, culture-based cervicovaginal assessment in late pregnancy may provide descriptive microbiological information beyond GBS detection [12,13]. Such data may help define the spectrum of non-GBS cervicovaginal findings and guide future studies evaluating their clinical relevance [3,14].

In this context, prospective descriptive data from routine late-pregnancy cervicovaginal cultures may help clarify the spectrum of non-GBS microbiological findings without implying replacement of guideline-standard GBS screening or direct clinical outcome prediction. Accordingly, this prospective observational study aimed to describe the distribution of culture-based cervicovaginal findings among third-trimester pregnant women, particularly focusing on non-GBS findings and *Candida* colonization. In addition, among women with evaluable cervicovaginal cultures, we explored differences in maternal demographic, obstetric, and selected microscopic findings according to culture-confirmed Candida colonization, and evaluated direct microscopic yeast detection using culture-confirmed Candida colonization as the reference standard.

## 2. Materials and Methods

### 2.1. Study Design and Population

This prospective observational study was conducted at a tertiary care center and included third-trimester pregnant women (≥28 gestational weeks) receiving antenatal care. Eligible participants were consecutively recruited during the study period. In total, 300 pregnant women underwent cervicovaginal culture sampling. This study was designed to characterize culture-based cervicovaginal microbiological findings in late pregnancy, with a particular emphasis on non-GBS findings and culture-confirmed *Candida* colonization. GBS positivity was recorded descriptively in the overall cohort. For the analyses of culture-confirmed *Candida* colonization, only samples reported as having fecal contamination were excluded. Women with GBS-positive cervicovaginal cultures were retained in the evaluable cohort because GBS and *Candida* colonization are not mutually exclusive. Exclusion criteria included multiple pregnancies, antibiotic use within the preceding two weeks, known immunosuppressive conditions, and refusal to provide informed consent.

### 2.2. Sample Collection and Microbiological Analysis

Cervicovaginal swab samples were obtained using sterile swabs and transported to the microbiology laboratory for culture-based assessment. The collected specimens were processed according to institutional culture-based microbiological protocols, and the microbiological results were interpreted by experienced microbiology specialists using conventional culture-based methods. As rectal specimens were not obtained, the GBS-related findings were interpreted as cervicovaginal culture results rather than as guideline-standard vaginal–rectal screening results [8]. Culture material was inoculated onto eosin methylene blue (EMB) agar, sheep blood agar, and Thayer–Martin agar and incubated for 48 h according to the institutional microbiology laboratory protocol. Colonies grown on culture media were evaluated using catalase and PYR (pyrrolidonyl arylamidase) tests, and GBS identification was performed using a Streptococcal Grouping test kit based on the Lancefield latex agglutination method. Cervicovaginal culture findings were classified as normal vaginal flora, GBS-positive culture, *Candida* colonization, other non-GBS bacterial isolates, or fecal contamination. GBS positivity was recorded when GBS was identified in the cervicovaginal culture. *Candida* colonization was defined as any culture report indicating the presence of *Candida albicans*, non-albicans *Candida*, *Candida* spp., or mixed normal vaginal flora with *Candida* species. Direct microscopic evaluation was performed using wet-mount preparations made from cervicovaginal swab material. The preparations were examined under light microscopy for the presence of clue cells, yeast forms, and *Trichomonas vaginalis*. No additional staining, KOH preparation, or molecular testing was performed as part of the direct microscopic evaluation. Microscopic findings, including clue cell positivity, direct microscopic yeast detection, and *Trichomonas vaginalis* positivity, were recorded separately.

### 2.3. Data Collection

Maternal demographic, obstetric, and baseline clinical data were obtained from medical records and standardized study forms. The recorded variables included maternal age, pre-pregnancy body mass index, gravidity, parity, gestational age at sampling, obstetric history, and routinely recorded baseline clinical characteristics. Baseline clinical characteristics were summarized descriptively and were not included in Candida-focused comparative analyses. The microbiological variables included cervicovaginal culture results, GBS positivity, culture-confirmed *Candida* colonization status, and microscopic findings, including clue cell positivity, direct microscopic yeast detection, and *Trichomonas vaginalis* positivity.

### 2.4. Statistical Analysis

Statistical analyses were performed using IBM SPSS Statistics version 24.0 (IBM Corp., Armonk, NY, USA). Descriptive statistics were used to summarize maternal demographic, obstetric, baseline clinical, microbiological, and microscopic variables. Categorical variables are presented as frequencies and percentages. Continuous variables are presented as the mean ± standard deviation or median with interquartile range, as appropriate. The overall distribution of culture-based cervicovaginal findings was evaluated in the entire cohort (*n* = 300). For exploratory comparisons according to culture-confirmed *Candida* colonization, only samples reported as having fecal contamination were excluded, leaving 298 women for analysis. Women with GBS-positive cervicovaginal cultures were retained in this analysis. Continuous variables were compared using the Mann–Whitney U test, and categorical variables were compared using Fisher’s exact test because of small expected cell counts. Direct microscopic yeast detection was not evaluated as an associated factor; instead, its diagnostic performance was assessed using culture-confirmed *Candida* colonization as the reference standard. Sensitivity, specificity, positive predictive value, negative predictive value, and accuracy were calculated from a 2 × 2 contingency table, and 95% confidence intervals were calculated using the Wilson method. Baseline clinical characteristics were summarized descriptively and were not included in *Candida*-focused comparative analyses. A two-sided *p*-value of <0.05 was considered to indicate statistical significance.

### 2.5. Sample Size Consideration

A sample size adequacy assessment was performed using G*Power version 3.1.9.7. For a chi-square test with 1 degree of freedom, assuming a medium effect size (w = 0.30), an alpha level of 0.05, and 95% power, the minimum required sample size was estimated as 145 participants. The planned and enrolled cohort of 300 women exceeded this minimum. After excluding samples reported as having fecal contamination, 298 women were included in the exploratory analyses of culture-confirmed *Candida* colonization. Women with GBS-positive cervicovaginal cultures were retained in this analysis because GBS and *Candida* colonization are not mutually exclusive. Because the study was primarily descriptive and was not powered to evaluate clinical risk factors for *Candida* colonization, analyses involving low-frequency microbiological findings were interpreted cautiously.

### 2.6. Ethical Considerations

The study protocol was approved by the Ethics Committee of the University of Health Sciences, Ankara Health Research and Application Center (approval date: 5 December 2019; approval number: 00128). Written informed consent was obtained from all participants before enrollment. The study was conducted in accordance with the principles of the Declaration of Helsinki.

## 3. Results

### 3.1. Study Population and Baseline Characteristics

A total of 300 third-trimester pregnant women (mean maternal age: 26.48 ± 5.67 years) were included in the study. The median gravidity and parity were 2.0 (1.0–3.0) and 1.0 (0.0–2.0), respectively. The mean gestational age at sampling was 34.85 ± 3.38 weeks, with a range of 28.00–41.43 weeks. With respect to obstetric history, 92 women (30.7%) were nulliparous, 138 (46.0%) had a history of vaginal delivery, 58 (19.3%) had a history of cesarean delivery, and 12 (4.0%) had a history of both vaginal and cesarean delivery. The baseline demographic and obstetric characteristics of the study population are summarized in Table 1.

### 3.2. Culture-Based Cervicovaginal Microbiological Findings

Normal vaginal flora was reported in 252 of 300 women (84.0%). *Candida* colonization was the most frequent non-GBS finding, identified in 35 women (11.7%). GBS was detected in the cervicovaginal cultures of seven women (2.3%). Other non-GBS bacterial isolates were detected in the cervicovaginal cultures of four women (1.3%), including Gram-negative bacteria, *Gardnerella vaginalis*, and *Staphylococcus aureus*. Samples reported as fecal contamination were observed in two cases (0.7%). Overall, non-GBS cervicovaginal findings were more frequently observed than GBS detection in cervicovaginal cultures, with *Candida* species representing the predominant non-GBS microorganisms. The distribution of culture-based cervicovaginal and microscopic findings is summarized in Table 2. Microscopic findings were evaluated separately from culture-based findings. Clue cells were identified in 20 women (6.7%), yeast was detected by direct microscopy in 12 women (4.0%), and *Trichomonas vaginalis* positivity was detected in six women (2.0%). Direct microscopic yeast detection was less frequent than culture-confirmed *Candida* colonization.

### 3.3. Associations with Culture-Confirmed Candida Colonization

For the analysis of variables associated with culture-confirmed *Candida* colonization, only samples reported as having fecal contamination (*n* = 2) were excluded. Women with GBS-positive cervicovaginal cultures were retained in the evaluable cohort because GBS and *Candida* colonization are not mutually exclusive. Accordingly, 298 women with evaluable cervicovaginal cultures were included in this analysis. Of these, 35 had culture-confirmed *Candida* colonization and 263 did not. No statistically significant differences were observed between women with and without culture-confirmed *Candida* colonization in terms of maternal age, gravidity, parity, gestational age at sampling, clue cell positivity, *Trichomonas vaginalis* positivity, or GBS-positive cervicovaginal culture. The median maternal age was 25.0 years in the *Candida*-positive group and 26.0 years in the *Candida*-negative group (*p* = 0.786). The median gravidity was 2.0 in both groups (*p* = 0.918), and the median parity was also similar between the groups (*p* = 0.875). Gestational age at sampling did not differ significantly between women with and without *Candida* colonization (35.57 weeks vs. 35.00 weeks, respectively; *p* = 0.751). Clue cell positivity was observed in 3 of 35 women (8.6%) with *Candida* colonization and in 16 of 263 women (6.1%) without *Candida* colonization, with no statistically significant association (*p* = 0.476). *Trichomonas vaginalis* positivity was detected in 2 of 35 women (5.7%) with *Candida* colonization and in 4 of 263 women (1.5%) without *Candida* colonization; this difference was not statistically significant (*p* = 0.149). GBS-positive cervicovaginal culture was observed in 7 of 263 women (2.7%) without *Candida* colonization and in none of the women with *Candida* colonization (*p* = 1.000).

Exploratory comparisons according to culture-confirmed *Candida* colonization are summarized in Table 3. Maternal age, gravidity, parity, gestational age at sampling, clue cell positivity, *Trichomonas vaginalis* positivity, and GBS-positive cervicovaginal culture did not differ significantly between women with and without culture-confirmed Candida colonization.

Direct microscopic yeast detection was evaluated as a diagnostic finding rather than as a factor associated with Candida colonization. Among 35 women with culture-confirmed Candida colonization, five had yeast detected by direct microscopy, and 30 were microscopy-negative. Among 263 women without culture-confirmed Candida colonization, six had yeast detected by direct microscopy, and 257 were microscopy-negative. Culture and microscopy results showed statistically significant concordance by Fisher’s exact test (*p* = 0.005), although direct microscopy demonstrated low sensitivity. Using culture-confirmed Candida colonization as the reference standard, direct microscopic yeast detection showed a sensitivity of 14.3%, a specificity of 97.7%, a positive predictive value of 45.5%, a negative predictive value of 89.5%, and an accuracy of 87.9% (Table 4).

Among women with culture-confirmed *Candida* colonization, *Candida albicans* and *Candida* spp. each accounted for 16 of 35 cases (45.7%), whereas non-albicans *Candida* accounted for three cases (8.6%) (Supplementary Figure S1).

## 4. Discussion

In this prospective observational study of third-trimester pregnant women, normal vaginal flora was the predominant cervicovaginal culture finding, whereas *Candida* colonization was the most frequent non-GBS finding. Culture-confirmed *Candida* colonization was not significantly associated with maternal age, gravidity, parity, or gestational age at sampling. Direct microscopic yeast detection was not interpreted as an associated factor; instead, it was evaluated as a diagnostic finding using culture-confirmed *Candida* colonization as the reference standard. This diagnostic performance analysis showed that direct microscopic yeast detection had low sensitivity but high specificity, indicating that a positive microscopic finding may support *Candida* detection, whereas a negative microscopic result does not exclude culture-confirmed colonization. Although GBS was infrequently detected in cervicovaginal cultures in the present cohort, this finding should be interpreted with caution. Current guideline-recommended GBS screening recommends combined lower vaginal and rectal sampling because this approach increases culture yield compared with cervical-only or vaginal-only sampling [8]. Conversely, the present study used culture-based cervicovaginal samples without rectal sampling. Therefore, GBS-related findings should be interpreted as cervicovaginal culture results rather than estimates of guideline-standard maternal GBS colonization prevalence [8,15]. Moreover, studies have shown that GBS detection is influenced by both specimen type and laboratory method, with molecular approaches generally yielding higher detection rates than conventional culture alone [16,17,18,19]. Within this culture-based cervicovaginal framework, non-GBS findings were more frequently observed than GBS findings, with *Candida* colonization representing the leading non-GBS culture-confirmed finding. Rather than challenging established GBS screening recommendations, these findings suggest that cervicovaginal cultures may provide additional descriptive microbiological information beyond GBS status, particularly regarding fungal colonization. The principal contribution of this study is not to propose cervicovaginal culture as an alternative to guideline-standard GBS screening or as a predictor of clinical outcomes, but to provide prospective descriptive data on the spectrum of culture-based cervicovaginal findings in late pregnancy and to show the limited sensitivity of wet-mount microscopy for culture-confirmed *Candida* colonization.

*Candida* colonization was the leading non-GBS finding in this cohort. Recent evidence suggests that vaginal *Candida* colonization during pregnancy may contribute to neonatal exposure and vertical transmission, supporting the relevance of documenting fungal colonization, even though maternal and neonatal outcomes were not evaluated in the present study [20]. *Candida* colonization and vulvovaginal candidiasis are commonly reported during pregnancy, although their frequency varies according to population characteristics, symptom status, gestational age, and diagnostic approach [21,22]. Recent microbiome-based data also suggest that pregnancy-associated vulvovaginal candidiasis may be characterized by gestational stage-specific dysbiosis and reduced *Lactobacillus* dominance [23]. Eskezia et al. reported vaginal *Candida* species in 30% of pregnant women receiving antenatal care, whereas GBS colonization was not detected in their cohort [24]. Weldegebreal et al. also reported a substantial burden of bacterial vaginosis and vaginal *Candida* colonization among pregnant women, highlighting that non-GBS vaginal findings remain clinically relevant in antenatal populations [25]. In the present cohort, *Candida albicans* and culture reports categorized as *Candida* spp. were equally represented among culture-confirmed *Candida* cases, whereas non-albicans *Candida* accounted for a smaller proportion. Because some isolates were reported only as *Candida* spp., detailed species-level interpretation is limited.

A key additional finding was the discordance between culture-confirmed *Candida* colonization and direct microscopic yeast detection. When culture-confirmed *Candida* colonization was used as the reference standard, direct microscopy showed low sensitivity but high specificity. This indicates that direct microscopic yeast detection may be useful when positive, but a negative wet-mount microscopic result does not necessarily exclude culture-confirmed *Candida* colonization. The low sensitivity observed in the present study may be partly related to the use of wet-mount preparations without KOH preparation, additional staining, or molecular testing, as well as to low fungal burden in colonized women. The discordant culture-positive/microscopy-negative findings may be explained by low fungal burden, the limited sensitivity of wet-mount microscopy, the absence of KOH preparation or additional staining, and the inability of direct microscopy to reliably detect low-level colonization. Conversely, culture-negative/microscopy-positive findings may reflect morphologic misclassification, nonviable fungal elements, sampling variability, or organisms that were not recovered under the applied culture conditions. Therefore, these discordant results should be interpreted as a limitation of routine wet-mount microscopy rather than as evidence against culture-confirmed colonization. This interpretation is consistent with previous evidence showing that the apparent prevalence of vaginal *Candida* colonization varies according to diagnostic method, with molecular methods detecting *Candida* more frequently than wet-mount or smear microscopy [26]. Therefore, direct microscopic examination may be informative when positive, but a negative microscopic result does not necessarily exclude culture-confirmed *Candida* colonization. No significant differences were detected in maternal age, gravidity, parity, or gestational age at sampling, clue cell positivity, or *Trichomonas vaginalis* positivity between women with and without culture-confirmed *Candida* colonization. These findings suggest that, within this third-trimester cohort, the evaluated maternal, obstetric, and selected microscopic variables did not distinguish women with culture-confirmed *Candida* colonization from those without colonization. Baseline clinical characteristics were summarized descriptively but were not included in *Candida*-focused comparative analyses because the study was not designed to evaluate clinical risk factors for *Candida* colonization. This may reflect the multifactorial nature of cervicovaginal colonization and the limited ability of routine demographic or obstetric variables alone to distinguish women with culture-confirmed Candida colonization. Studies evaluating vaginal candidiasis during pregnancy have emphasized the importance of species-level identification and antifungal susceptibility patterns, particularly because *Candida albicans* and non-albicans *Candida* species may differ in epidemiology, antifungal susceptibility, and clinical relevance [27]. Therefore, future studies should incorporate standardized fungal identification and, when clinically indicated, antifungal susceptibility testing.

The strengths of this study include its prospective observational design, the inclusion of a well-defined third-trimester cohort, and the concurrent evaluation of culture-based cervicovaginal findings and direct microscopic parameters. This study also provides a practical descriptive perspective by reporting the spectrum of microorganisms recovered from cervicovaginal samples collected during late pregnancy. In addition, direct microscopic yeast detection was evaluated using a diagnostic performance framework rather than as an associated factor, providing a more appropriate interpretation of culture–microscopy concordance. These findings may provide a basis for future studies incorporating symptom assessment, standardized sampling, molecular microbiome profiling, species-level fungal identification, and clearly defined maternal and neonatal outcomes.

### Study Limitations

This study has several limitations. First, the analysis was based on conventional culture and wet-mount microscopy, and molecular microbiome profiling was not performed. Therefore, the microbial community structure, relative abundance, and uncultivable microorganisms could not be evaluated. Second, direct microscopic evaluation was performed using wet-mount preparations without additional staining, KOH preparation, or molecular testing, which may have contributed to the low sensitivity of direct microscopic yeast detection. Third, symptom status, treatment data, prior vulvovaginal candidiasis, recent antifungal treatment, and clinically defined maternal or neonatal outcomes, including delivery outcomes, neonatal infection, and neonatal intensive care admission, were not part of the prespecified analytical scope of the present study and were therefore not evaluated. Accordingly, the findings should be interpreted as culture-based microbiological observations rather than as evidence of clinical consequences or maternal, clinical, or neonatal outcome-related associations. Fourth, baseline clinical characteristics, including pre-pregnancy body mass index and routinely recorded maternal comorbidities, were summarized descriptively only and were not included in Candida-focused comparative or multivariable analyses. Fifth, species-level Candida identification was incomplete because some isolates were reported as Candida spp. rather than being fully speciated. Therefore, detailed comparisons between Candida albicans and non-albicans Candida could not be performed, and antifungal susceptibility patterns were not evaluated. Finally, rectovaginal sampling was not performed; only cervicovaginal swab samples were obtained. Therefore, GBS detection in this cohort may have underestimated the true maternal GBS colonization rate, and the low GBS positivity rate observed in cervicovaginal cultures should be interpreted descriptively rather than as a guideline-standard vaginal–rectal GBS screening prevalence estimate.

## 5. Conclusions

In this prospective observational third-trimester cohort, *Candida* colonization was the most frequent culture-confirmed non-GBS cervicovaginal finding. Direct microscopic yeast detection showed low sensitivity but high specificity when culture-confirmed *Candida* colonization was used as the reference standard, suggesting that wet-mount microscopy alone may underestimate fungal colonization. Culture-confirmed *Candida* colonization was not significantly associated with the evaluated maternal, obstetric, or selected microscopic variables. Because symptom status, treatment implications, and maternal or neonatal outcomes were not evaluated, the clinical significance of asymptomatic *Candida* colonization cannot be determined from these data. Larger studies incorporating standardized sampling, species-level fungal identification, symptom assessment, molecular methods, and pregnancy outcomes are needed. These findings should be interpreted as descriptive microbiological observations and not as evidence of clinical outcome associations.

## Figures and Tables

**Table 1:** Baseline demographic, obstetric and clinical characteristics of the study population (*n* = 300).

Characteristic	Value
Demographic variables	
Maternal age, years, mean ± SD	26.48 ± 5.67
Pre-pregnancy body mass index, kg/m^2^, median (IQR)	21.5 (19.0–24.0)
Gravidity, median (IQR)	2.0 (1.0–3.0)
Parity, median (IQR)	1.0 (0.0–2.0)
Gestational age at sampling, weeks, mean ± SD	34.85 ± 3.38 (range, 28.00–41.43)
Obstetric history	
Nulliparous	92 (30.7)
Previous vaginal delivery	138 (46.0)
Previous cesarean delivery	58 (19.3)
Previous vaginal and cesarean deliveries	12 (4.0)
Clinical characteristics	
Diabetes mellitus	7 (2.3)
Gestational diabetes mellitus	23 (7.7)
Chronic hypertension	5 (1.7)
Hypertensive disorders of pregnancy	23 (7.7)
Epilepsy	5 (1.7)
Thyroid disease	6 (2.0)
Asthma	25 (8.3)

Values are presented as the mean ± standard deviation, median (interquartile range), range, or n (%), as appropriate. Percentages were calculated using the total study population (*n* = 300). Obstetric history categories are mutually exclusive. Hypertensive disorders of pregnancy included preeclampsia. Baseline clinical characteristics were obtained from routine antenatal records and summarized descriptively.

**Table 2:** Culture-based cervicovaginal and microscopic findings (*n* = 300).

Variable	*n* (%)
Culture-based cervicovaginal findings	
Normal vaginal flora only	252 (84.0)
*Candida* colonization (total)	35 (11.7)
*GBS*-positive cervicovaginal culture	7 (2.3)
Other non-GBS bacterial isolates *	4 (1.3)
Fecal contamination	2 (0.7)
Microscopic findings	
Clue cell positivity	20 (6.7)
Yeast detected by direct microscopy	12 (4.0)
*Trichomonas vaginalis* positivity	6 (2.0)

Values are presented as n (%). Percentages were calculated using the total study population (*n* = 300). GBS, Group B *Streptococcus*. * Other non-GBS bacterial isolates include Gram-negative bacteria, *Gardnerella vaginalis*, and *Staphylococcus aureus*. Fecal contamination was recorded separately and was not classified as a non-GBS bacterial isolate. Culture-based categories were mutually exclusive; microscopic findings were recorded separately from culture-based categories.

**Table 3:** Exploratory comparisons according to culture-confirmed *Candida* colonization among women with evaluable cervicovaginal cultures after exclusion of fecal contamination (*n* = 298).

Variable	*Candida*-Negative (*n* = 263)	*Candida*-Positive (*n* = 35)	*p* Value
Maternal age, years	26.0 (22.0–30.0)	25.0 (23.0–31.0)	0.786
Gravidity	2.0 (1.0–3.0)	2.0 (2.0–3.0)	0.918
Parity	1.0 (0.0–2.0)	1.0 (0.0–2.0)	0.875
Gestational age at sampling, weeks	35.00 (32.00–37.57)	35.57 (32.29–37.57)	0.751
Clue cell positivity	16 (6.1)	3 (8.6)	0.476
*Trichomonas vaginalis* positivity	4 (1.5)	2 (5.7)	0.149
GBS-positive cervicovaginal culture	7 (2.7)	0 (0.0)	1.000

Values are presented as medians (interquartile ranges) or n (%). The percentages are column percentages within each *Candida* colonization group. GBS, Group B *Streptococcus*. Only samples reported as having fecal contamination (*n* = 2) were excluded from this analysis. Women with GBS-positive cervicovaginal cultures were retained in the evaluable cohort. *p*-values were calculated using the Mann–Whitney U test for continuous variables and Fisher’s exact test for categorical variables.

**Table 4:** Diagnostic performance of direct microscopic yeast detection using culture-confirmed Candida colonization as the reference standard.

Diagnostic Measure	Value, % (95% CI)
Sensitivity	14.3 (6.3–29.4)
Specificity	97.7 (95.0–98.9)
Positive predictive value	45.5 (21.3–72.0)
Negative predictive value	89.5 (85.5–92.6)
Accuracy	87.9 (83.7–91.1)

Culture-confirmed Candida colonization status was used as the reference standard for this diagnostic performance analysis. The analysis included women with evaluable cervicovaginal cultures after exclusion of samples reported as fecal contamination (*n* = 298). Confidence intervals were calculated using the Wilson method. CI: confidence interval.

## Data Availability

The data underlying the findings of this study are available from the corresponding author upon reasonable request.

## Supplementary Materials

The following supporting information can be downloaded at: https://www.mdpi.com/article/10.3390/medicina62071389/s1, Figure S1 . Distribution of culture-reported Candida categories among women with culture-confirmed Candida colonization. Candida albicans and Candida spp. each accounted for 16 of 35 cases (45.7%), whereas non-albicans Candida accounted for 3 of 35 cases (8.6%).
