# Supplementary material for: Culture-Based Cervicovaginal Findings and Candida Colonization in Third-Trimester Pregnancy: A Prospective Observational Study
Source: Medicina (Kaunas). 2026 Jul 18;62(7):1389. doi: 10.3390/medicina62071389 (PMC13413755; doi:10.3390/medicina62071389)
Supplement: Supplementary file 1 [file medicina-62-01389-s001.zip › medicina-4364576-supplementary.pdf]

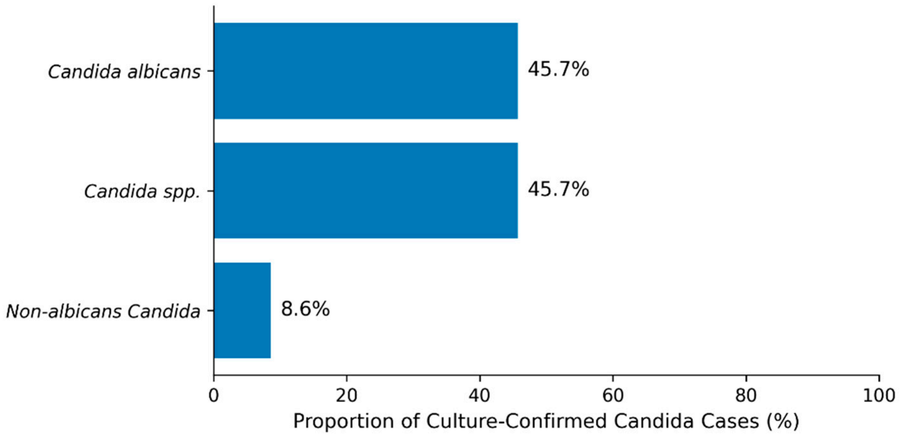

Supplementary Figure S1. Distribution of culture-reported *Candida* categories among women with culture-confirmed *Candida* colonization. *Candida albicans* and *Candida* spp. each accounted for 16 of 35 cases (45.7%), whereas non-albicans *Candida* accounted for 3 of 35 cases (8.6%).
